# Genetic diversity and chemical profiling of different populations of *Convolvulus pluricaulis* (convolvulaceae): an important herb of ayurvedic medicine

**DOI:** 10.1007/s13205-014-0227-8

**Published:** 2014-06-18

**Authors:** Showkat Hussain Ganie, Zahid Ali, Sandip Das, Prem Shankar Srivastava, Maheshwar Prasad Sharma

**Affiliations:** 1 Department of Botany, Jamia Hamdard, Hamdard Nagar, New Delhi, 110062 India; 2 Department of Biotechnology, Jamia Hamdard, Hamdard Nagar, New Delhi, 110062 India; 3 Department of Botany, University of Delhi, New Delhi, 110007 India

**Keywords:** *Convolvulus pluricaulis*, Genetic diversity, HPLC, Kaempferol, RAPD

## Abstract

*Convolvulus pluricaulis* Choisy, commonly known as “Shankhpushpi”, is an ayurvedic medicinal plant recommended as a brain tonic to promote intellect and memory, eliminate nervous disorders and to treat hypertension. Because of increasing demand of the drug, this plant species has been over-exploited. As a consequence, many unrelated plants are being sold by the crude drug dealers in India in the name of “Shankhpushpi”. Information on its existing gene pool is currently lacking. We developed molecular (Random Amplification of Polymorphic DNA) and chemical (high performance liquid chromatography) markers that could distinguish the genuine plant species from its adulterants. Molecular characterization confirmed higher genetic variation at inter-zonal level as compared to intra-zonal populations. A total of 37 reproducible amplicons were generated of which 22 were polymorphic. The number of amplicons was in the range of 6–11 and genetic distance for the studied primers ranged from 0.07 to 0.34. Fifty nine per cent polymorphism was obtained across different geographical locations. Dendrogram studied through unweighted pair group method of arithmetic analysis differentiated all the genotypes into two major clusters, Cluster I had the single population of Rajasthan and Cluster II was represented by genotypes of Delhi, Haryana, Madhya Pradesh and Rajasthan. The Kaempferol content ranged from 0.07 to 0.49 mg/g and Delhi population was the highest accumulator.

## Introduction

The indigenous plant-based systems of medicine (Ayurveda, Siddha and Unani) have been in existence for several centuries and continue to serve humanity for infinite time to come. The use of medicinal plants registered a decline with the development of synthetic drugs and antibiotics, but the toxicity and harmful side effects of synthetic drugs have again brought medicinal plants to the forefront of health care system. However, overpopulation and over-exploitation of medicinal plants, particularly in the developing countries, have caused extensive damage to the medicinal plants wealth. Therefore, characterization and conservation of medicinal plants is the need of hour. Herbal medicinal materials are traditionally identified by their organoleptic or microscopic characteristics, including size, shape, colour, odour, flavour, texture and other physical properties. However, these methods are considered to be subjective because the morphological and chemical characters can be influenced by the environment and changes in different developmental stages. Molecular markers are not influenced by environmental factors; tests can be carried out at any time during any stage of plant development; they have the potential of existing in unlimited numbers, covering the entire genome. Moreover, a small amount of sample is sufficient for analysis. A number of PCR-based molecular markers have been used for detecting polymorphism at DNA level. Among them, random amplification of polymorphic DNAs (RAPD) gained much popularity because of its simplicity, non-requirement of prior information of nucleotide sequence and can be performed with very small amount of genomic DNA. Random amplification of polymorphic DNA technique has been successfully employed for the estimation of genetic diversity; some of which include *Plantagoovata* (Singh et al. 2009), *Ricinuscommunis* (Gajera et al. 2010), *Jatrophacurcas* (Zhang et al. 2011), *Curcumalonga* (Singh et al. 2012), *Aloevera* (Nejatzadeh-Barandozi et al. 2012) and *Clitoriaternatea* (Yeotkar et al. 2012). In addition to DNA markers, phytochemical markers high performance liquid chromatography (HPLC) play a role to portray genetic variability and authentication of medicinally important plants (Li et al. 2008). Hence, molecular and chemical characterization can go hand in hand rather than in isolation because such studies would be quite helpful for conservation strategies and the selection of population containing maximum content of active compound.

*Convolvulus pluricaulis* (Family: Convolvulaceae) is a prostrate spreading wild herb. In India it has a narrow distribution found in plains of Punjab, Uttar Pradesh, Haryana, Rajasthan, Bihar and Chota Nagpur (Sethiya and Mishra 2010). The plant is used in Ayurveda to cure various ailments. It is recommended as a brain tonic to promote intellect and memory, eliminate nervous disorders and to treat hypertension (Bala and Manyam 1999); is anti-helmintic, good in dysentery, hair tonic, cures skin ailments and reduces high blood pressure (Rai 1987). The leaves are recommended for depression and mental disturbance (Singh and Mehta 1977). The herb has been widely used to treat nervous disorders, similar to the use of kava kava (*Piper methysticum*) and valerian (*Valeriana officinalis*) prescribed by American herbalists (Husain et al. 2007).

The plant shows the presence of alkaloids, glycosides, coumarins and flavonoides (Bhowmik et al. 2012). One of the flavonoid in *C*. *pluricaulis* is Kaempferol (Andrade et al. 2012); it is reported to have significant biological activity being used for chemo-preventive purposes like inhibition of cell growth (Jin et al. 1995), induction of apoptosis (Jin et al. 1995; Braig et al. 2005; Chen and Kong 2005; Niering et al. 2005) and inhibition of proteasome activity (Chen and Kong 2005).

Being found in open waste lands, the natural populations of this herb has depleted to a great extent due to over-exploitation and habitat degradation, especially in urban areas, and therefore, need protection. This species, therefore, appears well suited for study using molecular and chemical markers to determine its genetic diversity. The present study is based on RAPD and HPLC analyses, of the materials collected from different locations in India.

## Materials and methods

**Fig. 1:**
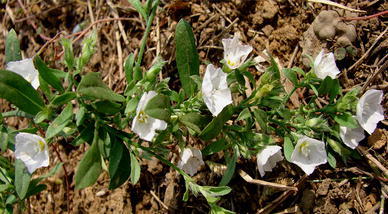
*Convolvulus* *pluricaulis*

The samples of C. *pluricaulis* were collected from Aravali foothills (Delhi and Kurukshetra, Haryana), Gangetic Plains (Lucknow, Uttar Pradesh), Arid zone (Jodhpur, jaipur, Udaipur, Rajasthan) and Vindhyachal (Bhopal, Madhya Pradesh). Whole herb was collected, transferred to sealable polythene bags and transported to the laboratory within 10–24 h depending upon the distance from the collection site. The samples were subjected to stringent method of botanical identification (Fig. 1); voucher specimens of the same were prepared and are kept in the Herbarium, Department of Botany, Hamdard University, New Delhi, 110062. The identified specimens were compared with authenticated voucher specimens preserved in the herbarium of National Institute of Science Communication and Information Resources (NISCAIR). The lyophilized leaves were used for DNA isolation. A part of the plant material was dried at 40 °C for HPLC analysis.

### DNA isolation and RAPD assay

The modified CTAB protocol of Doyle and Doyle (1990) and purification kit (HiPurA) were used to isolate DNA from the leaves. The leaves (1 g) were pulverized to fine powder using liquid nitrogen in a chilled mortar and pestle followed by the addition of 100 mg of PVP (insoluble) and 10 ml pre-heated CTAB buffer (CTAB 2 %, 2 M Sodium Chloride, 100 mM Tris HCl- pH 8, 20 mM EDTA). The slurry was transferred into autoclaved 50 ml centrifuge tube and incubated at 60 °C for 1 h. After incubation, the tubes were kept at room temperature for 20 min and 10 ml of chloroform, isoamyl alcohol (CHCl_3_: IAA, 24:1) was added and mixed carefully for 15 min. The content was centrifuged at 8,000 rpm for 15 min at 15 °C. The upper phase was collected in fresh autoclaved centrifuge tubes to which 10 µg/ml of RNAase was added and the tubes were incubated at 37 °C for 30 min. To inactivate RNAase A, 10 ml CHCl_3_: IAA (24:1) was added and the content was centrifuged at 8,000 rpm for 15 min at 15 °C. The upper phase was transferred again into autoclaved centrifuge tube and 0.5 vol of 3 M Potassium acetate (pH 5.2) was added. To precipitate the DNA, two volumes of chilled absolute ethanol was used and the tubes were kept at **−**20 °C for 2 h. It was recentrifuged at 8,000 rpm for 15 min at 4 °C. The supernatant was discarded and the pellet was washed with 70 % ethanol, air dried and dissolved in 250 µl of sterile water. The DNA obtained was not ideal for PCR analysis and therefore was purified by DNA purification kit (HiPurA, India) according to manufacturer’s instructions.

The polymerase chain reaction was carried out in 15 µl reaction volume containing 50 ng DNA, 0.5 units Taq DNA polymerase, 1.66 mM MgCl_2_, 30 pmol 11-mer primers, 200 µM of each dNTPs, 1 × Taq polymerase buffer. The final volume was made up with sterile MilliQ water. The amplifications were carried out in DNA thermal cycler (Eppendorf, Germany). The PCR amplification conditions for RAPD consisted of initial step of denaturation at 94 °C for 4 min, 35 cycles of denaturation at 94 °C for 1 min, annealing at 35 °C for 1 min, extension at 72 °C for 2 min, followed by final extension at 72 °C for 10 min. The amplified DNA was loaded on 1.2 % agarose gel in 0.5 × TBE buffer containing 10 µl of EtBr (10 mg/ml) and photographed using gel documentation system (UVP, Germany). Lambda DNA *EcoR* 1- *Hin*d 111 double digest was used as molecular marker (Bangalore Genei, Bangalore, India) to know the size of the fragments. Twenty-five 11-mer RAPD primers of OPN and G series, purchased from Eurofins MWG Operon, Germany), were screened. The data analysis was carried out by band scoring of well-marked amplified fragments.

### Data and statistical analysis

PCR products were scored for the presence (1) or absence (0) irrespective of band intensity since each product of identical molecular weight was supposed to represent a single locus. Genetic analysis was carried out using Nei genetic similarity index (Nei and Li 1979) using the formula *F*_*xy*_ = 2*n*_*xy*_/(n_*x*_ + n_*y*_), where *n*_*xy*_ is the number of common RAPD fragments shared by two samples and *n*_*x*_ and *n*_*y*_ are the total number of bands scored in each sample. The genetic distance was calculated using Hillis and Mortiz equation (1990), *D* = 1−F, where “*F*” is species similarity. The dendrogram was constructed using the NTSYS-pc software (Numerical taxonomy and multivariate system) (Rohlf 1993).

### HPLC analysis

The leaf powder (400 mg) was first defatted by pre-extraction with 20 ml chloroform and refluxed for 1 h with 30 ml 95 % aqueous methyl alcohol (MeOH) and 9 ml of 25 % hydrochloric acid. After filtration with Whatman paper, the samples were extracted twice with 20 ml of MeOH for 10 min. The combined hydrolysates were diluted with MeOH to 100 ml and filtered through syringe filter (0.45 μm). Each extract was injected in triplicate. The kaempferol reference standard (Sigma) in different concentrations was prepared in parallel to generate standard curve for quantification. Kaempferol quantification was made by comparing its retention time of the peak area with the Kaempferol peak area from the standard. The retention time of standard Kaempferol was 2.13. HPLC analysis conditions were: Waters 600E HPLC system equipped with 125 × 4 mm C18 column with PDA detector 996 and auto sampler 2701. The mobile phase was 2 % acetic acid and methanol: acetic acid: water (18:18:1 v/v) at 1 ml/min constant flow rate, 35 °C column temperature and 370 nm wavelength for detection. The injection volume was 20 μl.

## Results and discussion

In order to study the diversity at DNA level in *C. pluricaulis*, 25 primers were used for RAPD analysis. Only five primers generated clear and reproducible bands, with the size range of 0.5–2.0 kb. A total of 37 bands (Table 1), with an average of 7.4 bands per primer, were produced. Number of polymorphic bands per primer ranged from 01 to 09 with an average of 4.8 polymorphic bands per primer. The mean percentage of polymorphic bands was 59.45. Such average polymorphism might be due to non-effective gene flow, low fecundity, low pollen flow, local selection procedure (environment and struggle for existence), inbreeding systems (Loveless and Hamrick 1984), biotic factors like human interference, habitat destruction and commercial exploitation (Vijay et al. 2009). The earlier work carried out on similar aspects lend support to our study that also recorded average polymorphism at individual provenance/genotype level in *Lycoris longituba* (65. 96 %, Deng et al. 2006), *Asparagus racemosus* (54.92 %, Vijay et al. 2009), *Typha angustifolia* (71 %, Na et al. 2010).

**Table 1:** RAPD data and percentage of polymorphism in *C. pluricaulis*

S. no	Primer code	Nucleotide sequence (5^′_^3^′^)	Total no. of bands	Polymorphic bands	% Monomorphism	% Polymorphism
1	OPN-01	CCTCAGCTTGG	11	09	18.82	81.18
2	OPN-02	AACCAGGGGCA	07	05	28.58	71.42
3	OPN-04	GGACCGACCCA	06	06	0.00	100.00
4	OPN-09	TTGCCGGCTTG	07	01	85.72	14.28
5	G-01	ATGCTCTGCCC	06	01	83.34	16.66
	Average		37	22	40.55	59.45

**Fig. 2:**
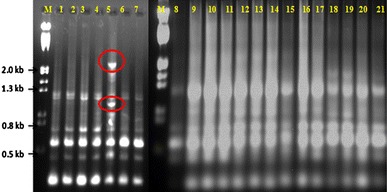
RAPD fingerprint obtained with OPN-09 primer in different accessions of *C. pluricaulis: M* Marker (λ DNA digested with *Hind* III and *Eco*R I); *1*, *2* Kurukshetra Campus (Haryana); *3*, *4* Arjun Herbal Park Kurukshetra (Haryana); *5* Jodhpur (Rajasthan); *6*, *7* Lucknow (Uttar Pradesh); *8*–*11* Hamdard Campus (Delhi); *12*–*15* Bhopal (Madhya Pradesh); *16*–*18* Udaipur (Rajasthan); *19*–*21* Jaipur (Rajasthan). *Circles* represent region-specific bands

**Fig. 3:**
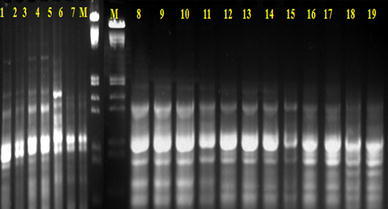
RAPD fingerprint obtained with G-01 primer in different accessions of *C. pluricaulis: M* Marker (λ DNA digested with *Hind* III and *Eco*R I); *1*, *2* Kurukshetra Campus (Haryana); *3*, *4* Arjun Herbal Park Kurukhshetra (Haryana); *5* Jodhpur (Rajasthan); *6*, *7* Lucknow (Uttar Pradesh); *8*–*11* Hamdard Campus (Delhi); *12*–*15* Bhopal (Madhya Pradesh); *16*–*17* Udaipur (Rajasthan); *18*–*19* Jaipur (Rajasthan)

The primers OPN-04, OPN-01 and OPN-02 generated highest percentage polymorphisms (100, 81 and 71.42, respectively), and the lower polymorphisms (16.66 and 14.28 %) were obtained with G-01 and OPN-09 (Figs. 2, 3). Of the total bands generated, 15 were monomorphic across all the genotypes. The genetic similarity and distance was in the range of 0.66–0.93 and 0.07–0.34, respectively (Table 2).

**Table 2:** Average genetic distance in different genotypes of *C. pluricaulis*

Genotype	01	02	03	04	05	06	07	08
01	–	0.07	0.27	0.14	0.09	0.08	0.23	0.18
02	0.07	–	0.25	0.15	0.11	0.16	0.20	0.26
03	0.27	0.25	–	0.30	0.34	0.29	0.31	0.30
04	0.14	0.15	0.30	–	0.17	0.16	0.14	0.20
05	0.09	0.11	0.34	0.17	–	0.07	0.11	0.17
06	0.08	0.16	0.29	0.16	0.07	–	0.11	0.13
07	0.23	0.20	0.31	0.14	0.11	0.11	–	0.14
08	0.18	0.26	0.30	0.20	0.17	0.13	0.14	–

01 Haryana (Kurukshetra Campus), 02 Haryana (Arjun Herbal Park-Kurukshetra), 03 Rajasthan (Jodhpur), 04 Uttar Pradesh (Lucknow), 05 Delhi (Hamdard Campus), 06 Madhya Pradesh (Bhopal), 07 Rajasthan (Udaipur), 08 Rajasthan (Jaipur)

Population-specific bands generated through different primers represented the identification marks for various genotypes. The unique bands of 0.8 and 0.6 kb amplified by primer OPN-01, one band of 0.35 kb amplified by OPN-02 and bands of 2.1 and 1.0 kb developed by primer OPN-09 are specific to Rajasthan genotypes. The drug Shankhpushpi is being equated with three different plant species (*Clitoriaternatea*, *Convolvuluspluricaulis* and *Evolvulusalsinoides*); studies based on relative efficacy and usage suggest that *C. pluricaulis* can be considered as the actual source, while *E. alsinoides* and *C. ternatea* as the alternative sources of Shankhpushpi (Nair et al. 1997). In addition to these three plants, different unrelated drugs are being sold in crude drug markets of India in the name of Shankhpushpi (Singh and Viswanathan 2001). The specific bands obtained, therefore, will help distinguish the authentic drug from its substitutes and adulteraterants. Molecular markers have been used in the identification of herbal drugs (Rout 2006; Rivera-Arce et al. 2007; Irshad et al. 2009; Heubl 2010; Ganie et al. 2012).

**Fig. 4:**
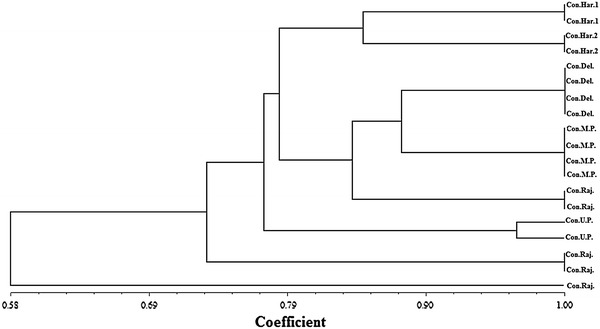
Dendrogram showing the similarity coefficients in different accessions of *C. pluricaulis*. *Har*. *1* (Haryana, Kurukshetra Campus), *Har*. *2* (Haryana, Arjun Herbal Park, Kurukshetra), *Del* (Delhi, Hamdard Campus), *M. P*. (Madhya Pradesh, Bhopal), *Raj.* (Rajasthan—Udaipur, Jaipur and Jodhpur), *U. P*. (Uttar pradesh, Lucknow)

The similarity coefficients were used to generate a tree for cluster analysis using the UPGMA method. The resulting dendrogram differentiated two major clusters. First represented a solitary genotype of Jodhpur (Rajasthan) and the second, the genotypes of Kurukshetra (Haryana), Delhi, Bhopal (Madhya Pradesh), Udaipur, Jaipur (Rajasthan) and Lucknow (Uttar Pradesh) (Fig. 4). The genotypes of Delhi and Bhopal shared highest level of similarity coefficient (0.87). The sample collected from Jodhpur (Rajasthan) had the highest degree of divergence and shared a similarity coefficient of only 0.58 with the rest of the genotypes. The intra-zonal diversity was maximum among Rajasthan populations followed by Haryana populations whereas in other samples, intra-zonal diversity was negligible. The clustering obtained was not region specific, thus showing the lack of any defined population structure in this species. Similar finding have been reported for *Prunus cerasifera* (Ayanoglu et al. 2007) and *Punica granatum* (Jbir et al. 2008). However, Ali et al. (2013) reported well-structured clustering in *Clitoria ternatea* and Belaj et al. (2010) for Andalusian and most Catalonian olive cultivars. Other than Rajasthan genotypes that remained scattered all over the dendrogram, all other genotypes formed perfect clusters by remaining confined within their respective clades.

**Fig. 5:**
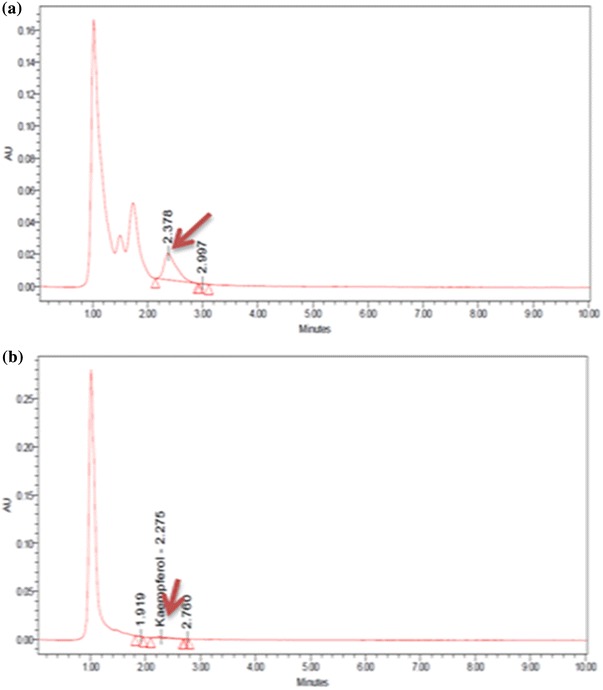
Chromatograms showing kaempferol content of *C. pluricaulis* collected from (**a**) Delhi, (**b**) Madhya Pradesh

The HPLC analysis also showed considerable variation in kaempferol content in different genotypes investigated. The results are presented in Table 3. Delhi population was found to be the highest accumulator (0.495 ± 0.014 mg/g dry wt) of kaempferol, followed by populations of Haryana (0.375 ± 0.002 mg/g dry wt), and the least kaempferol content was observed in Madhya Pradesh (0.076 ± 0.002 mg/g dry wt) populations (Fig. 5). The plant collection was made in different geographical locations; variation found, therefore, might be due to different environmental conditions. However, variation in kaempferol content could also be due to intra-specific nucleotide sequence differences. Hence, both genetic as well as environmental factors may be responsible for variation in kaempferol. The variation could be correlated by Sabu et al. (2001) equation: VK = VG + VE, where VK is the variation in kaempferol content, VG is the genetic variance and VE is the environmental variance. The average climate of a particular geographical region remains almost constant; therefore, our aim was to screen the different populations of *C*. *pluricaulis* on the basis of kaempferol content. Furthermore, secondary metabolite content generally increases with the maturity and hence, the genotypes were collected at the flowering stages.

**Table 3:** Kaempferol content in different samples of *C. pluricaulis*

S. no	Plant samples	Amount of kaempferol (mg/g dry wt) ± SE
1	Delhi, Hamdard Campus	0.495 ± 0.014
2	Haryana, Kurukshetra	0.375 ± 0.002
3	Uttar Pradesh, Lucknow	0.184 ± 0.007
4	Rajasthan, Jodhpur	0.279 ± 0.003
5	Rajasthan, Jaipur	0.23 ± 0.002
6	Rajasthan, Udaipur	0.19 ± 0.001
7	Madhya Pradesh, Bhopal	0.076 ± 0.002

Though simultaneous molecular and chemical characterization is rare in medicinal plants, there are some reports where both the techniques have been taken into consideration. Boszormenyi et al. (2009) correlated RAPD profile with the essential oil composition among sage cultivars. Han et al. (2008) studied the genetic and chemical profile in different populations of *Fructus xanthii* in China. Similarly genetic and the terpenoid profile of *Zataria multiflora* collected from different locations in Iran have been analyzed (Hadian et al. 2011).

On the basis of differences in kaempferol content, the populations of *C. pluricaulis* could be divided into three groups. The first group comprised the accessions of Delhi and Haryana (0.495, 0.375), the group II, genotypes of Rajasthan (Jodhpur, Jaipur and Udaipur) and Uttar Pradesh (0.279, 0.23, 0.19 and 0.184), and the group III represented accession from Madhya Pradesh (0.076). Other than kaempferol, a number of medicinally important secondary metabolites of *C. pluricaulis* have also been isolated of which scopoletin has been quantified by HPLC using different solvent systems (Kapadia et al. 2006; Upadhyay et al. 2013). The scopoletin content obtained by Upadhyay et al. (2013) was highest in hydro-alcoholic extract (0.1738 %) followed by methanolic extract (0.0932 %) and aqueous extract (0.0435 %). Our results declared that the herb contained less kaempferol in comparison to scopoletin. Delhi populations being rich in kaempferol may potentially be multiplied and used on a large scale for commercial cultivation. The kaempferol content could further be increased by tissue culture and transformation studies. Our next focus is to isolate the kaempferol from *C*. *pluricaulis* and determine its anti-cancer activity using rat models.

A comparative molecular and chemical analysis of different population of *C. pluricaulis* was achieved in this work. RAPD technology could be efficiently used to demonstrate genetic relationships among different populations of *C. pluricaulis*. By analysing the genetic and chemical profiling, it is possible to identify the elite population. This information could be employed for devising strategies for authentication and conservation of *C. pluricaulis*.
